# P02 Assessment of the appropriateness of antibiotic prescribing in an acute UK hospital using a national audit tool: a single-centre retrospective cohort study

**DOI:** 10.1093/jacamr/dlac053.002

**Published:** 2022-05-31

**Authors:** Neil Powell, Kathleen Bamford, Junior Doctor RCHT PHE Audit Team

**Affiliations:** Royal Cornwall Hospital NHS Trust, UK

## Abstract

**Background:**

Defining the gold standard for appropriate antibiotic prescribing is challenging due to the subjective nature of evaluating quality in prescribing. In 2017 the UK Government Scientific Advisory Committee on Antimicrobial Prescribing, Resistance and Healthcare Associated Infection (APRHAI) used the Delphi process to define appropriate antibiotic prescribing, and developed an audit tool that supports patient-level assessment of antibiotic appropriateness in NHS hospitals.

**Objectives:**

To quantify excess antibiotic use in a 750-bed acute hospital in the south-west of England (bottom 20th centile on PHE Fingertips for total antibiotic prescribing), and to determine where in the prescribing process the opportunity to safely reduce antibiotic use is: (A) initiation, (B) the pre-72 h review, or (C) course length optimization.

**Methods:**

The EPMA system was used to identify patients discharged from medical specialties in August 2020 who had received at least one dose of antibiotic. Patients were grouped by discharging medical specialty, as follows: acute medicine, elder care, renal, endocrine, gastroenterology, respiratory and cardiology. Medical notes for 25% of discharges from each specialty were requested and audited against the APRHAI audit tool. A junior doctor completed the audit form for each patient with the case then discussed by two infection specialists until consensus was reached. Excess days of antibiotic therapy (DOTs) were calculated for each of the three timepoints (A–C).

**Results:**

In total, 184 of 647 (28%) patients discharged from medical specialties were audited: 85 female, 99 male, median age 69 years (IQR 60–82), total DOTs 1658 of which 403 (24%) were excess. Excess DOTs by timepoint: A (initiation) 112 (28%), B (pre-72 h review) 184 (46%), C (course length) 107 (27%). Ninety-four of 187 (50%) of patients recorded zero excess DOTs.

**Conclusions:**

We did not identify any excess DOTs in half of all patients initiated on antibiotics. However, in the other half we identified a significant proportion of excess DOTs with most excess DOTs at the pre-72 h review.

